# A Retrospective Evaluation of Snake Envenomation in Dogs in South Korea (2004–2021)

**DOI:** 10.3390/toxins14080565

**Published:** 2022-08-18

**Authors:** Jeong-Min Lee, Joong-Hyun Song, Kun-Ho Song

**Affiliations:** College of Veterinary Medicine, Chungnam National University, Daejeon 34134, Korea

**Keywords:** snake, envenomation, dogs, antivenom, South Korea

## Abstract

Snake envenomation is a medical emergency capable of causing local and systemic complications. However, information on venomous snakebite in dogs in South Korea is scarce. In this study, fifty-nine dogs treated at a private veterinary clinic from 2004 to 2021 were retrospectively studied. The aim was to characterize the demographics, elapsed time between snakebite and veterinary clinic presentation, laboratory findings, clinical signs, treatments, adverse reactions to antivenom, and prognosis of venomous snakebite. Snakebite was mostly observed between 12 p.m. and 5 p.m. from April to October. On the days of envenomation, the weather conditions were mostly cloudy, followed by rain/precipitation, and least frequently fair weather. Grassland was the most common incident location, and leashed dog walking was the most frequent activity when snakebite occurred. The main local symptoms were edema, hemorrhagic discharge, cutaneous erythema, ulceration, and necrosis. Major systemic clinical signs were tachypnea, tachycardia, altered mentation, ptyalism, and hypotension. Based on the time interval between snakebite and presentation at the veterinary clinic, two groups were defined: <4 h (Group 1, 49.2%) and ≥4 h (Group 2, 50.8%). Systemic inflammation was more frequently observed in Group 2. The level of C-reactive protein at presentation (*p* = 0.036) and the highest-level during hospitalization (*p* = 0.023) were significantly elevated in Group 2 (≥4 h). The dogs in Group 2 displayed more frequent muscle damage (increased creatine kinase) than the dogs in Group 1, and a higher level of creatine kinase was associated with delayed (≥4 h) presentation after snakebite (*p* = 0.003). All of the dogs were treated symptomatically, and 34 dogs (58%) received antivenom. Treatment with antivenom showed no adverse reactions in this study. All of the treated dogs recovered. One dog was euthanized without any treatment due to respiratory distress, hypotension, and cost constraints. In conclusion, this study provides baseline information on venomous snakebite in dogs in South Korea. The prognosis was excellent, especially when the dogs were treated within 4 h.

## 1. Introduction

Snake envenomation is a significant public health issue for humans and animals in numerous countries. Every year, snakebite envenoming kills or maims approximately 100,000 and 400,000 people, respectively [1,2]. In South Korea, an average of 3020 snake envenomation victims are reported annually according to data from South Korea’s Health Insurance Review & Assessment Service, and the World Health Organization (WHO) database describes 101 incidents of casualties from snakebite envenomation in South Korea over 20 years from 1995 to 2015 [3,4]. Despite such medical significance, information on snakebite envenomation in the field of veterinary medicine in South Korea is limited.

There are around 3000 snake species worldwide, with about 600 being venomous [5]. South Korea has approximately 16 species of snakes, including four clinically significant venomous snakes [6,7,8]. Except for *Rhabdophis tigrinus*, belonging to the Colubridae family, the venomous snakes in South Korea belong to the Viperidae family [7]. The Viperidae family has been reported to account for 96.6% of snakebite cases in South Korea when the species was identifiable. This is followed by *Gloydius ussuriensis*, *Gloydius brevicaudus*, and *Gloydius intermedius*. It has also been reported that in 94.1% of cases, patients were unable to identify the genus level of the venomous snake [4,6,9]. Among the Gloydius species, *Gloydius ussuriensis* and *Gloydius brevicaudus* are frequently encountered species due to their wide-ranging habitats from lowlands to mountains [4]. *Gloydius intermedius* is reported comparatively infrequently as it generally lives in mountains 500 m above sea level, making contact with people difficult [6].

Snake venom is one of the most complex natural toxins, comprising 50–200 proteinaceous components [10,11]. Clinically relevant major toxins in snake venoms, such as phospholipase A_2_ (PLA_2)_, snake venom serine proteases (SVSPs), snake venom metalloproteinases (SVMPs), and three-finger peptides (3FTxs), are directly and indirectly responsible for the various pharmacological effects appearing in snakebite patients [12]. The effects of snake venoms can be pharmacologically classified into three types: hemotoxic, neurotoxic, and cytotoxic [13,14]. First, hemotoxic venom has hemostatic effects and cardiovascular effects [11]. Reduction of blood coagulability, impairment of blood vessels, and thrombosis can be caused by an affected hemostatic system, and cardiovascular effects could induce substantial low blood pressure [11,15]. Second, neurotoxic envenoming targets multiple sites of the neuromuscular junction causing interference with neurotransmission that could result in neuromuscular weakness [11,16]. Last, cytotoxic envenoming kills cells by modulating membrane-bound enzyme activity, disrupting platelet aggregation, and causing depolarization in neurons and the heart cell’s excitable membrane [17].

The prompt administration of antivenom is the primary treatment for venomous snakebite [18,19,20]. Snake antivenom should ideally be administered within four hours of a bite but can still be effective after two or more weeks to reverse systemic envenoming, especially in the case of hemostatic abnormalities [2,18,21]. In humans, WHO guidelines recommend antivenom treatment in the following circumstances: (1) systemic envenomation, including cardiovascular abnormalities, hemostatic abnormalities, neurotoxic signs, acute kidney injury, hemoglobinuria, myoglobinuria, or laboratory evidence of systemic envenoming; (2) the rapid expansion of swelling or local swelling occupying more than 50% of the bitten limb without a tourniquet; (3) development of an enlarged lymph node draining the bitten limb [2]. Additionally, supportive treatment for canine snake envenomation can include fluid therapy, pain management, antibiotic prophylaxis, respiratory support, and surgical debridement [22]. In South Korea, Kovax^®^ freeze-dried *Gloydius brevicaudus* antivenom (KOREAVACCINE Co., Ltd., Seoul, South Korea; 6000 units/vial) is the main treatment for snakebite in humans [6,20]. However, veterinary reports on the efficacy and safety of antivenom administration in South Korea are scarce.

We report detailed clinical cases of snake envenomation in dogs in South Korea, examining the demographics, meteorological conditions at the time of envenomation, incident location, activity at the time of snakebite, time and date, laboratory findings, local and systemic clinical signs, treatments, and outcomes.

## 2. Results

### 2.1. General and Epidemiological Data

This retrospective study focused on 74 dogs that had been treated for snakebite envenomation at a private veterinary clinic in South Korea between 2004 and 2021. Among the 74 cases, 15 were excluded from the study for having incomplete medical records, not developing symptoms, or displaying clinical signs inconsistent with snake envenomation. A total of 59 records satisfied the inclusion criteria.

The main demographics of the dogs bitten by venomous snakes are shown in Table 1. Among the 59 canine patients included in this study, 27 (46%) were male (11 sexually intact and 16 neutered), and 32 (54%) were female (16 sexually intact and 16 spayed). The mean age was 3.7 years (range 0.4–15 years, median 3.4 years), and the mean body weight was 14.42 kg (range 1.9–48 kg, median 13.5 kg). The most common breeds affected were mix-breeds (*n* = 17; 29%), Maltese (*n* = 4; 7%), Beagle (*n* = 3; 5%), Dachshund (*n* = 3; 5%), Welsh Corgi (*n* = 3; 5%) and Labrador Retriever (*n* = 3; 5%).

**Figure 1:**
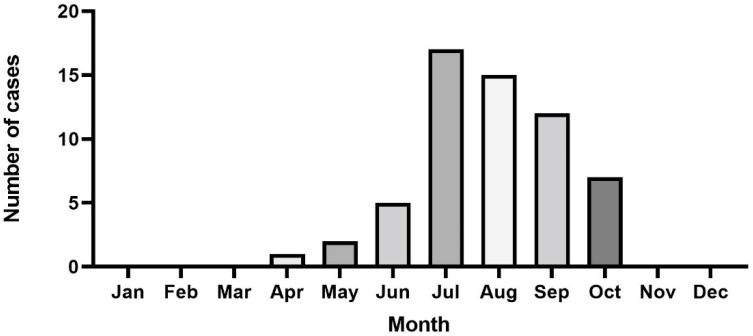
Distribution of months of presentation for the 59 dogs.

The circumstances, meteorological conditions, incident locations, and dog activity at the time of snake envenomation are reported in Table 2. Medical records indicated that snake envenomation occurred mostly after 12 p.m. and before 5 p.m. (40.6%; n = 24), followed by after 5 p.m. and before 9 p.m. (23.7%, n = 14). The median interval time between snakebite and veterinary clinic admittance was 4 h (IQR: 2–10). Most of the snake envenomation occurred in the month of July (28.8%), followed by August (25.4%) and September (20.3%). No snake envenomation was detected from November to March, corresponding with the snake hibernation period (Figure 1).

Meteorological conditions on the days of envenomation are described in Table 2. The most frequent weather was cloudy (54.2%), followed by rain/precipitation (32.2%), and fair weather (13.6%). The average relative humidity was 67.2%, and cloud cover was 5.25. The average temperature was 24.5 °C, with an average daily low of 20.4 °C and an average daily high of 29.13 °C. The characteristics of the weather indicate that snake envenomation in the dogs occurred more frequently during cloudy and rain/precipitation weather conditions than during fair weather, which is consistent with the average cloud cover and relative humidity. The dogs were most frequently bitten in grassland (71.2%), followed by back yards (18.6%) and mountains (10.2%). When the dogs were bitten, the majority were being walked on a leash (67.8%). Two of the dogs were envenomated while guarding a property in a backyard, which was discovered by viewing footage recorded by surveillance cameras installed at the properties.

### 2.2. Clinical Data

**Figure 2:**
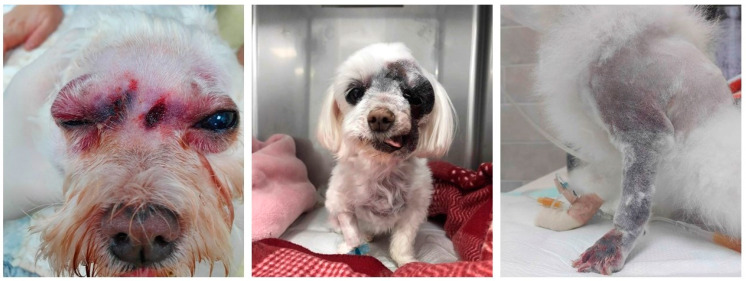
Local edema, hemorrhagic discharge with cutaneous erythema observed in a dog with snake envenomation.

Bite sites, length of hospitalization, and clinical details of the patients are shown in Table 3. Fang marks were observed in 41 dogs (69%). Most of the bites were in the head/muzzle region (69%), followed by the forelimb (19%), hindlimb (5%), and neck (5%). The thorax region was the least common bite site. The local symptoms of envenomation included swelling near the site of envenomation (100%), hemorrhagic discharge (59.3%), cutaneous erythema (39%), ulceration (28.8%), and necrosis (18.6%) (Figure 2). Systemic clinical signs included tachypnea (63.2%), tachycardia (33.3%), altered mentation (20%), ptyalism (13.6%), and hypotension (11.1%). Rectal temperature was obtainable in 42 dogs (72%), with the median temperature being 38.7 °C (IQR: 38.5–39.3). Clinical signs showed no significant difference between the dogs in each group.

A statistical comparison of the laboratory findings for the two groups is presented in Table 4. Forty-eight patients had a complete blood count (CBC) performed. The predominant laboratory abnormality at presentation was thrombocytopenia, which was observed in 29% of the patients. Furthermore, leukocytosis (17%) and anemia (12%) were notable hematological abnormalities at admission. The median value of prothrombin time (PT) was 10.05 (IQR: 10.05–12.00), with prolonged PT being detected in 17% of the dogs. The median value of activated partial thromboplastin time (aPTT) was 72.50 (IQR: 33.65–96.05), and prolonged aPTT was detected in 58% of the dogs. The dogs that showed prolonged PT also showed prolonged aPTT in this study. Significant differences were not observed in CBC, electrolyte values, or coagulation examination between the patients examined <4 h or ≥4 h after envenomation. A biochemical value measuring alanine aminotransferase (ALT), alkaline phosphatase (ALP), aspartate aminotransferase (AST), total bilirubin, blood urea nitrogen (BUN), and creatinine did not show remarkable differences between the two groups; however, the serum creatine kinase (CK) level was substantially higher in Group 2 (*p* < 0.01). In Group 2, the level of C-reactive protein (CRP) at presentation *(p* = 0.036) and the highest-level during hospitalization (*p* = 0.023) were statistically significant. Additionally, the level of glucose (*p* = 0.012) was significantly different between the patients examined within 4 h and the patients examined 4 h or longer after envenomation.

### 2.3. Treatment and Outcome

**Figure 3:**
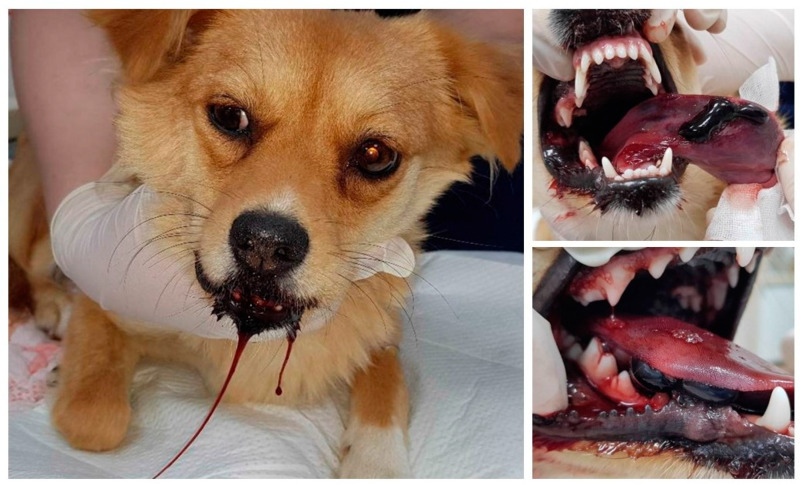
Hemorrhagic blister and edema at the tongue in a dog bitten by snake envenomation.

Thirty-four dogs (58%) received antivenom therapy shortly after presentation at the clinic. Among the patients administered antivenom, 27 were subjected to an antivenom skin test, with all returning negative test results. Adverse reactions, including pyrogenic reactions and facial swelling, were not observed in the dogs that received antivenom. Twenty-four dogs (42%) did not receive antivenom due to the owner’s rejection or because the dogs only showed mild local swelling without systemic signs of envenomation; however, all were treated symptomatically. All patients in this study received bite site cleaning and dressing, 40 (67.8%) received fluid therapy, and 8 (13.6%) received oxygen therapy. Chlorpheniramine maleate was administered to 44 (74.6%), and dexamethasone sodium phosphate was administered to 28 (47.5%). Forty-eight patients (81.4%) received antimicrobials, including ceftezole sodium, enrofloxacin, ampicillin, and amoxicillin/clavulanic acid. Norepinephrine and dobutamine were administered to three patients (5.1%) with hypotension. Concerning pain, 50 dogs (84.7%) were treated with analgesics, including tramadol, butorphanol, ketamine, and gabapentin. Additional therapies included the administration of famotidine to 41 dogs (69.5%), maropitant to 16 (27.1%), and tranexamic acid to 8 (13.6%). Surgical debridement was performed on two dogs (3.3%). A nasoesophageal feeding tube was placed in one dog that had difficulty eating due to hemorrhagic blisters on its tongue after being bitten by a snake (Figure 3).

Fifty-eight of the 59 patients (98%) treated recovered successfully. One dog was euthanized due to severe respiratory distress, hypotension, depressed mentation, and cost constraints. The median lengths of hospitalization for Group 1 and Group 2 were 2.1 ± 2.57 days and 2.5 ± 2.57 days, respectively. Statistically significant differences were not observed (*p* = 0.495).

## 3. Discussion

Snake envenomation is a frequently confronted medical emergency in humans in South Korea [3,4,6,20]. In veterinary medicine, however, the clinical characteristics and treatment of venomous snakebites are insufficiently documented [23]. Thus, the present study provides a comprehensive insight into snakebite in dogs in South Korea. In this study, the majority of the dogs were young adults less than four years of age, with a similar proportion of female and male dogs experiencing snake envenomation. This may be related to the similar inquisitive nature and playfulness of male and female dogs [24,25]. We evaluated the meteorological conditions, clinicopathological findings, treatments, and prognoses in 59 venomous snakebite cases.

Since a venomous snakebite is a medical emergency, the most effective way to manage snake envenomation is to prevent bites from occurring. Accordingly, WHO recommends understanding the characteristics of local snakes. This includes knowing the times of day, night, year, and the meteorological conditions during which snakes are most active because active hours and seasons vary according to species and habitat [14,26,27]. Daily and seasonal snake activity patterns have been described for many snake species in different regions, but few studies have been undertaken in South Korea [28]. In this study, we discovered that dogs were bitten more frequently during afternoon hours and warm seasons. Many factors influenced this result, including the snakes and envenomated dogs; however, the number of snakebites peaking in the afternoon suggests that snakes in South Korea are most active during the hottest part of the day. Furthermore, the dogs were mostly bitten between May and October, the hottest months in South Korea [4,9].

Changes in seasonal temperatures are generally presumed to influence a snake’s activity and lifecycle because of its poikilothermic nature, i.e., snakes are affected by ambient temperature and humidity [28,29,30]. Indeed, it is well established that snake activity positively correlates with temperature and humidity [28,31]. Snakes become more active as temperatures increase, especially when the mean temperature is greater than 23°C, with maximum activity occurring at around 30°C [28,32]. Humidity is another factor determining snake activity as moving in dry conditions may lead to water evaporation and desiccation [33]. In one study, snakes were found to be two times more active during periods of high humidity compared to low humidity [28]. The relative humidity was 67.22 ± 9.29 % in our study.

Snake envenomation was more likely to happen on days with precipitation and cloudy weather compared to fair weather in this study. The correlation between snakebite incidence and weather has been reported to vary by region. For example, drought conditions have been discovered to increase the incidence of snakebite in Costa Rica and Alabama [30,34]. In contrast, snakebite incidence has been found to increase after precipitation in California [35]. In addition, factors including the locations and activities of the patients could affect snakebite incidence. Grassland was the location where the dogs were most frequently bitten, which is consistent with the ecological features of the two common species *Gloydius ussuriensis* and *Gloydius brevicaudus* that live in lowland grassland [6]. Most of the dogs were bitten while being walked on a leash. In general, many owners believe that walking a dog on a leash may protect their dogs from snakes. However, considering the results of this study, it is important for owners to pay close attention even when using a leash. Consequently, as numerous factors, including meteorological conditions, the availability of prey, and the locations of dogs, affect the incidence of snakebite, understanding the characteristics of local snakes is necessary. Bites mainly occurred on the head and muzzle, followed by the forelimbs, indicating a high incidence of encounters between dogs and snakes. These findings support studies in Sweden, South Africa, and Sri Lanka [25,36,37]. On arrival at the veterinary clinic, 20% of the dogs displayed an affected mental status. Fang marks, one or two puncture wounds typically indicating a poisonous snakebite, were recognized on 69.5% of the dogs upon examination at admission [38].

Human snakebite victims are classified as presenting early (less than 4 h after bite) or late (more than 4 h after bite) [39]. Victims who receive treatment late are likely to experience more severe envenomation resulting in longer hospitalization and greater expense [39]. The median interval time between being bitten and receiving treatment was 4 h (IQR: 2–10) in our study. More specifically, 49.2% of the patients were examined at the veterinary clinic in less than 4 h, and 10.2% were admitted 24 h after snake envenomation.

The clinical signs of snake envenomation stem from the various peptides and proteins in the venom, with the composition varying according to species, age, location, and diet [11,12,15,17,40]. The *Gloydius* species, which is most likely to cause snake envenomation in South Korea, has various peptides and proteins causing coagulopathy [20,41]. Some of the components identified include brevinase, salmosin, halyxin, halysase, salmorin, colobin, and saxatilin which affect platelets, coagulation factors, coagulation products, and blood vessels, possibly leading to venom-induced consumption coagulopathy (VICC) [42,43]. Besides the coagulopathic components of venom, various other proteins and peptides influence the hemotoxic, cytotoxic, and neurotoxic effects of snake envenomation; hence, all of the patients in this study experienced local complications, including edema, hemorrhagic discharge, change in color, ulceration, and necrosis at the bite site 2 [44]. A significant proportion of the dogs suffered from tachypnea (63.2%), and further systemic signs, including tachycardia, hypotension, altered mentation, increased salivation, and cyanosis were observed. Necrosis was more frequent in Group 2 (26.7%) compared to Group 1 (10.3%). However, including necrosis, there were no statistically significant differences between the two groups regarding clinical symptoms in this study. 

A complete blood count at admission revealed that thrombocytopenia was a predominant abnormality. Although the exact mechanism is yet to be completely understood, it is speculated that both activation and inhibition of platelets by venom components contribute to venom-induced coagulopathy by exhausting platelets, resulting in marked thrombocytopenia [45]. Other laboratory abnormalities, including leukocytosis and anemia, were observed in less than 20% of patients. Laboratory results of coagulation examination detected coagulopathy with prolonged PT in 17% of the dogs and prolonged aPTT in 59%. Coagulopathy induced by snake venom, called VICC, is frequently detected in patients bitten by Asian vipers [41]. VICC is characterized by low fibrinogen, elevated D-dimer levels, and prolonged PT and aPTT in animals [43,46]. In humans, VICC is further classified into partial or complete VICC based on the severity of coagulopathy, but classification of VICC has not been determined in dogs [8,47]. As in human patients in South Korea, clinical deterioration induced by VICC was not observed in this study [8].

The findings of this study indicate significant differences between the patients in Group 1 and the patients in Group 2 regarding levels of serum creatine kinase (CK), C-reactive protein (CRP), and glucose. Elevated CK values were observed in approximately 65% of the envenomated dogs. CK is considered a marker of transient muscle cell damage [36]. It is predominantly located in skeletal muscle, the myocardium, the intestine, and the brain [48]. However, as the main source of serum CK stems from skeletal muscle, elevated levels of CK in serum generally indicate muscle injury or muscle stress [49]. In one study, CK values were an indicator of myonecrosis induced by snake envenomation, as a change in serum CK corresponded to necrotic intensity [50]. In our study, the serum CK level was remarkably higher in Group 2 (1050.75 ± 718.50) compared to Group 1 (333.30 ± 366.62), demonstrating that the time elapsed between bite and clinic admittance had a significant association with the serum CK level. Except for one patient (2%) that displayed mild azotemia, 98% of the dogs showed no evidence of renal failure.

Systemic inflammation induced by snake envenomation, defined as C-reactive protein >35 mg/L within 24 h of presentation [51], was found in 35% of the dogs. It is well established that levels of CRP rise as a result of inflammation, trauma, or infection within 4 to 6 h and peak at around 24 h [52,53]. Many other reports in humans and dogs have described increases in blood CRP concentration after snake envenomation [53,54,55]. In our study, CRP concentration in blood at admission was remarkably lower in Group 1. Higher CRP values at admission and the highest values during hospitalization were associated with delayed hospital presentation (≥4 h). Mild hyperglycemia, commonly reported in dogs and humans with snakebite envenoming [56,57], was also observed in this study. Numerous reports have demonstrated that acute illness or injury can result in transient stress hyperglycemia [58,59]. The mild hyperglycemia frequently observed in Group 2 was probably the result of the venom circulating longer and the delay in receiving adequate treatment for stress and pain following the snakebite [60].

Currently, intravenous administration of antivenom is the only standardized treatment for snake envenomation [2,6,8]. Antivenom contains polyclonal antibody fractions or antibodies that bind to circulating snake toxins [61]. This binding forms venom-antivenom complexes that block the venom target site or restrict the movement of the venom molecules in circulation [61,62]. The time between snake envenomation and antivenom injection is critical for effective treatment. Indeed, it is recommended that antivenom be administered within 4 h of snakebite for a more positive prognosis, but antivenom may still be effective even after several days when systemic envenoming persists, particularly in the case of hemostatic abnormalities [2,18]. Three types of antivenoms, including F(ab’)_2_ divalent fragments, whole IgG molecules, and monovalent Fab fragments, are produced by manufacturers [63]. The whole IgG antivenom, Kovax^®^ freeze-dried *Gloydius brevicaudus* antivenom (KOREAVACCINE Co., Ltd., South Korea; 6000 units/vial) is the sole antivenom administered in South Korea [6,20]. In this study, Kovax^®^ antivenom was administered to 58% (34/59) of the dogs. There were no deaths after antivenom administration. Identification of the genus level of the venomous snake was possible in only two cases (3.3%); one attributed to *Glyodius brevicaudus* and the other attributed to *Glyoidius intermedius*. In South Korea, among 3009 human snakebite reports, the genus level of the snake was identifiable in only 179 cases (5.9%) comprising 173 cases of *Gloydius* and six cases of *Rhabdophis tigrinus* envenomation [4]. Considering the difficulty in identifying snakes, the guidelines for snakebite management in South Korea recommend administering freeze-dried *Gloydius brevicaudus* antivenom to humans with unknown snakebite envenomation, as the Viperidae family accounts for 96.6% of snakebite reports [64]. In this study, 16 dogs received antivenom within 4 h of snakebite, while 18 received antivenom more than 4 h after snake envenomation. There were no significant differences regarding local complications or systemic clinical signs between the dogs receiving antivenom within 4 h and the dogs receiving antivenom after 4 h. Furthermore, there was no significant difference in the median length of hospitalization between the dogs treated with antivenom within 4 h and the dogs treated with antivenom later, possibly due to differences in the severity of each victim affected by the quantity and toxicity of the venom. Generally, the quantity and toxicity of venom is affected by the season, age, size, time since previous discharge, and motivation [1]. Among the 59 envenomated dogs, two received surgical debridement on the wound site due to severe necrosis. These dogs received supportive treatment including glucocorticoids, antihistamine, antibiotics, and analgesics within 4 h of envenomation, but antivenom was not administered due to financial constraints. Each dog was reexamined at 7 days and 10 days following snake envenomation and received surgical debridement, finally recovering well without complications. It has been speculated that antivenom has limited effectiveness in treating local tissue damage because its large molecular characteristic makes it unable to penetrate local tissue [63]. However, a recent study has revealed that antivenom can reach injured tissue sites, but reduced efficacy may result from endogenous proinflammatory mediators already activated before the administration of antivenom [63]. Hence, a short interval between snakebite and antivenom treatment is important for effective treatment, preferably within 4 h as C- reactive protein, creatine kinase, and glucose could reflect local tissue damage [49,59,65].

Adverse reactions and complications associated with antivenom have been reported differently in each country. The rate of adverse reactions to antivenom could result from poor quality and manufacturing control by manufacturers [19]. In humans, adverse reaction rates are around 1.5% in Europe, 2.4–8% in Japan, and 4.1% in South Korea [20]. These findings suggest that the antivenoms used in those countries are of high quality. In our study, neither acute reactions nor delayed reactions were observed in the dogs following antivenom administration. The use of premedication such as catecholamines, glucocorticoids, and histamine H1 antagonists is generally misconceived to prevent adverse reactions to antivenom by practitioners [66]. Recently, only adrenaline has shown meaningful results in clinical trials as an effective premedication for antivenom [66]. Moreover, in the case of anaphylaxis after antivenom administration, epinephrine injection should be considered as the treatment of choice. Antihistamines, corticosteroids, and fluid therapy should not be substituted for epinephrine [67]. The manufacturer’s instructions for antivenom use in South Korea state that a skin test should be performed before administration [64]. However, the use of skin tests has been questioned by one study that demonstrated a skin test showing 98.5% specificity but 17.5% low sensitivity, which could lead to misinterpreting the results [68]. Therefore, in veterinary medicine, further studies are necessary to assess the efficacy and safety of antivenom and skin tests in dogs in South Korea.

As in other retrospective studies, this study has some limitations: First, this study included only the results from a single private veterinary clinic in South Korea, and data review was limited by the available medical research, restricting generalizability. Second, the sample size was small, and dry-bite patients could not be included in this study because in most cases the genus level of the snake could not be identified, meaning that the authors were unable to differentiate venomous snakebites from non-venomous snakebites. Third, follow-up coagulation examination was not performed and the efficacy of antivenom was not evaluated. Fourth, refusal of the owner’s treatment meant that some of the dogs in this study did not receive adequate treatment including antivenom. Consequently, further multi-center prospective studies with larger groups and standardized treatment protocols not limited by finances are necessary.

## 4. Conclusions

This study shows that snake envenomation occurs more frequently in afternoons between May and October in South Korea. Additionally, cloudy weather and rainy days are more frequently associated with venomous snakebite. The envenomated dogs were treated symptomatically, with antivenom administered to 58%. Among the envenomated dogs, 98.3% recovered successfully. In conclusion, this study provides baseline information on snake envenomation in dogs in South Korea; however, further prospective, and controlled studies are necessary to substantiate the findings.

## 5. Materials and Methods

### 5.1. Study Design

This retrospective observational study was conducted at a private veterinary clinic. The population of this study comprised client-owned dogs. In total, 74 dogs were bitten by venomous snakes over a period of 18 years from January 2004 to December 2021. All medical records needed for patient evaluation were obtained from an electronic chart program (E-friends; pnV, Jeonju, South Korea).

### 5.2. Inclusion and Exclusion Criteria

Patients were included in this retrospective study if they received snake antivenom, if there was a strong suspicion of a venomous snakebite based on information obtained from the dog’s owner (e.g., the dog was observed being bitten or interacting with a venomous snake), or if patients exhibited clinical signs compatible with the diagnosis of snake envenomation such as swelling, hemorrhagic discharge, or changes in color with or without visible fang marks around the suspected bite location. Records of poisonous snakebites in which no symptoms developed and records of non-poisonous snakebites were excluded from the study. In addition, dogs were excluded if clinical signs were inconsistent with snake envenomation or if complete medical records were unavailable.

### 5.3. Determining the Indication of Antivenom Administration

The decision to administer antivenom to the patients was determined by the attending veterinarians based on the clinical status and species of snake. Rapid extension of swelling, enlarged lymph nodes of the bitten limbs, dark brown urine, muscle pain, neurotoxic signs, signs of haemostatic abnormalities, signs of cardiovascular abnormalities, and supporting abnormal laboratory findings of systemic envenomation were indicated for antivenom. For the patients showing only minor swelling with absence of systemic signs, antivenom was not indicated. In addition, *Rhabdophis tigrinus* envenomation was not indicated for antivenom, but for bites from unknown species showing signs of envenomation, antivenom was administered in accordance with human snakebite guidelines in South Korea [64].

### 5.4. Data Variables

The following data were obtained from medical records for analyses: history, estimated elapsed time between snakebite and hospital examination, clinical signs at admission, sex, age, breed, body weight, physical examination findings, mental status, localization of bites, treatments, length of hospitalization, outcome during hospitalization, and other laboratory tests results if available. Information regarding the meteorological conditions at the snakebite locations was downloaded from the Korea Meteorological Administration database (www.weather.go.kr, accessed on 6 July 2022). Further verification was obtained through telephonic interviews and medical records.

### 5.5. Statistical Analysis

Based on the time elapsed from snake envenomation to examination, two groups were defined: less than 4 h (Group 1) and more than 4 h (Group 2). All statistical analyses were performed using Microsoft Excel (version 16.0, 2019; Microsoft Corporation, Redmond, WA, USA), Prism Software (version 9.3.1, 2022; GraphPad Software, San Diego, CA, USA), and Statistical Package for the Social Sciences for Windows (version 27.0, 2021; Statistical Products and Service Solutions Inc., Chicago, IL, USA). Continuous variables were summarized using means ± standard deviations (SD). Median with interquartile range (IQR) was used to present the time interval between snakebite and treatment at the veterinary clinic. Chi-squared test or Fisher’s exact test was performed to examine relationships between categorical variables (Table 3). Independent t-test was performed to compare the means between two groups. A *p*-value of less than 0.05 was considered to be statistically significant (Table 3 and Table 4). 

## Figures and Tables

**Table 1:** Demographics of envenomated dogs.

Variable	Total	Group 1	Group 2
Gender			
Male	11 (18.6)	7 (24.1)	4 (13.3)
Neutered male	16 (27.1)	8 (27.6)	8 (26.7)
Female	16 (27.1)	8 (27.6)	8 (26.7)
Neutered female	16 (27.1)	6 (20.7)	10 (33.3)
Age (year)	3.78 ± 2.69	3.30 ± 2.12	4.24 ± 3.11
Weight (kg)	14.42 ± 9.89	15.16 ± 11.11	13.70 ± 8.69
Breed			
Mix-breeds	17 (28.8)	8 (27.6)	9 (30.0)
Maltese	4 (6.8)	2 (6.9)	2 (6.7)
Beagle	3 (5.1)	-	3 (10.0)
Dachshund	3 (5.1)	2 (6.9)	1 (3.3)
Welsh corgis	3 (5.1)	1 (3.4)	2 (6.7)
Labrador retrievers	3 (5.1)	2 (6.9)	1 (3.3)
Others	26 (44.1)	14 (48.3)	12 (40.0)
Total	59 (100.0)	29 (100.0)	30 (100.0)
N (%), Mean ± Standard Deviation (SD)			

**Table 2:** Characteristics and meteorological features of the case day.

Variable	Total	Group 1	Group 2
Time of day			
Morning (7 a.m.–12 p.m.)	11 (18.6)	7 (24.1)	4 (13.3)
Afternoon (12 p.m.–5 p.m.)	24 (40.7)	11 (37.9)	13 (43.3)
Evening (5 p.m.–9 p.m.)	14 (23.7)	7 (24.1)	7 (23.3)
Night (9 p.m.–7 a.m.)	10 (16.9)	4 (13.8)	6 (20.0)
Interval between bite and the admittance to the veterinary clinic			
<4 h	29 (49.2)	29 (100.0)	-
4–24 h	24 (40.7)	-	24 (80.0)
≥24 h	6 (10.1)	-	6 (20.0)
Mean ± SD	14.28 ± 37.49	1.88 ± 0.76	26.27 ± 50.08
Median(IQR)	4.0 (2.0–10.0)	2.00 (1.25–2.25)	9.00 (4.75–20.25)
Meteorological Condition			
Fair Weather	8 (13.6)	3 (10.3)	5 (16.7)
Cloudy Weather	32 (54.2)	20 (69.0)	12 (40.0)
Rain/Precipitation Weather	19 (32.2)	6 (20.7)	13 (43.3)
Meteorological Factor			
Temperature: Daily high	29.13 ± 5.14	28.79 ± 5.23	29.47 ± 5.12
Temperature: Daily low	20.40 ± 5.59	20.40 ± 5.98	20.40 ± 5.28
Temperature: Daily average	24.45 ± 5.21	24.32 ± 5.47	24.57 ± 5.02
Cloud cover	5.25 ± 2.88	5.20 ± 3.07	5.30 ± 2.73
Relative humidity	67.22 ± 9.29	67.82 ± 9.79	66.63 ± 8.90
Snakebite incident location			
Grassland	42 (71.2)	21 (72.4)	21 (70)
Mountain	6 (10.2)	3 (10.3)	3 (10.0)
Backyard	11 (18.6)	5 (17.2)	6 (20.0)
Activity at the time of snakebite			
Free run	17 (28.8)	6 (20.7)	11 (36.7)
Leashed walk	40 (67.8)	21 (72.4)	19 (63.3)
Guarding property	2 (3.4)	2 (6.9)	-
Total	59 (100.0)	29 (100.0)	30 (100.0)
N (%), Mean ± SD			

**Table 3:** Summary of bite site, length of hospitalization and clinical details of envenomated dogs.

Variable	Total	Group 1	Group 2	*p*
Mental				0.893
Alert	44 (80.0)	21 (80.8)	23 (79.3)	
Depressed	11 (20.0)	5 (19.2)	6 (20.7)	
Length of hospitalization (days)	2.37 ± 2.57	2.14 ± 2.68	2.60 ± 2.49	0.495
Bitesite				0.213 ^†^
Head/muzzle	41 (69.5)	23 (79.3)	18 (60.0)	
Lt. forelimb	6 (10.2)	1 (3.4)	5 (16.7)	
Lt. hindlimb	2 (3.4)	0 (0.0)	2 (6.7)	
Neck	3 (5.1)	2 (6.9)	1 (3.3)	
Rt. forelimb	5 (8.5)	2 (6.9)	3 (10.0)	
Rt. hindlimb	1 (1.7)	1 (3.4)	0 (0.0)	
Thorax	1 (1.7)	0 (0.0)	1 (3.3)	
Fang mark	41 (69.5)	22 (75.9)	19 (63.3)	0.296
Clinical signs				
Local edema	59 (100.0)	29 (100.0)	30 (100.0)	-
Ulceration	17 (28.8)	7 (24.1)	10 (33.3)	0.436
Cutaneous erythema	23 (39.0)	8 (27.6)	15 (50.0)	0.078
Hemorrhagic discharge	35 (59.3)	16 (55.2)	19 (63.3)	0.524
Necrosis	11 (18.6)	3 (10.3)	8 (26.7)	0.108
Tachypnea	24 (63.2)	10 (62.5)	14 (63.6)	0.943
Tachycardia	11 (33.3)	4 (26.7)	7 (38.9)	0.458
Hypotension	4 (11.1)	1 (6.3)	3 (15.0)	0.613 ^†^
Altered mentation	12 (20.7)	5 (17.9)	7 (23.3)	0.607
Ptyalism	8 (13.6)	6 (20.7)	2 (6.7)	0.145 ^†^
Lethargy	9 (15.3)	4 (13.8)	5 (16.7)	1.000 ^†^
Respiratory distress	2 (3.4)	0 (0.0)	2 (6.7)	0.492 ^†^
N (%), Mean ± SD				

^†^ Fisher’s exact test.

**Table 4:** Clinicopathological findings in 59 dogs presenting with snake envenomation.

Variable	Reference Range	Group 1		Group 2		*p*
		N	Mean ± SD	N	Mean ± SD	
Electrolytes						
Sodium	140–150 mEq/L	21	144.38 ± 5.13	21	143.10 ± 6.71	0.490
Potassium	3.9–4.9 mEq/L	21	4.02 ± 0.32	21	4.04 ± 0.37	0.909
Chloride	109–120 mEq/L	21	110.05 ± 5.42	21	110.24 ± 5.86	0.913
Blood						
Hemoglobin (admission)	13.1–20.5 g/dL	24	16.79 ± 3.00	24	16.20 ± 3.02	0.505
Hemoglobin (lowest)	13.1–20.5 g/dL	24	14.07 ± 3.76	24	12.77 ± 3.70	0.233
WBC (admission)	5.05–16.76 10^9^ cells/L	24	12445 ± 4407	24	15373 ± 5631	0.051
WBC (highest)	5.06–16.76 10^9^ cells/L	24	17,106 ± 11156	24	18,567 ± 6219	0.578
Hematocrit (admission)	37.3–61.7%	24	50.27 ± 8.32	24	48.98 ± 9.27	0.613
Platelet (admission)	148–484 10^9^ cells/L	24	285.25 ± 147.18	24	286.08 ± 242.59	0.989
Platelet (highest)	148–484 10^9^ cells/L	24	316.13 ± 142.87	24	331.25 ± 254.80	0.801
Platelet (lowest)	148–484 10^9^ cells/L	24	228.88 ± 151.25	24	220.75 ± 236.72	0.888
Coagulation examination						
PT	5–15 s	5	26.00 ± 35.80	7	16.01 ± 15.01	0.518
aPTT	15–45 s	5	67.74 ± 32.42	7	98.76 ± 113.38	0.570
Chemistry						
ALT	17–78 U/L	24	69.04 ± 104.39	24	65.38 ± 60.64	0.882
ALP	47–254 U/L	22	298.23 ± 630.29	22	286.05 ± 316.13	0.936
AST	17–78 U/L	23	72.30 ± 190.98	20	71.09 ± 50.27	0.978
CRP (admission)	0–10 mg/L	13	24.81 ± 45.67	18	70.00 ± 62.95	0.036 *
CRP (highest)	0–10 mg/L	13	28.46 ± 45.81	18	77.69 ± 62.94	0.023 *
Glucose	75–128 mg/d	22	115.45 ± 26.43	20	136.05 ± 23.88	0.012 *
Cholesterol	111–312 mg/d	12	200.42 ± 97.64	15	151.33 ± 58.46	0.143
Bilirubin (admission)	0.1–0.5 mg/d	21	1.03 ± 3.53	20	2.02 ± 6.65	0.555
Bilirubin (highest)	0.1–0.5 mg/d	20	1.67 ± 5.53	21	2.19 ± 6.45	0.782
CK	49–166 U/L	10	333.30 ± 366.62	16	1050.75 ± 718.50	0.003 **
BUN	9.2–29.2 mg/d	24	20.13 ± 7.21	24	21.06 ± 8.80	0.690
Creatinine	0.4–1.4 mg/dL	24	0.69 ± 0.20	24	0.77 ± 0.34	0.360
NH3	16–75 μg/d	17	58.76 ± 35.89	17	68.35 ± 40.17	0.468

* *p* < 0.05, ** *p* < 0.01.

## Data Availability

The data presented in this study are available in this article.
